# First person – Lara Cantarero

**DOI:** 10.1242/bio.059931

**Published:** 2023-04-07

## Abstract

First Person is a series of interviews with the first authors of a selection of papers published in Biology Open, helping researchers promote themselves alongside their papers. Lara Cantarero is first author on ‘ Differential effects of mendelian *GDAP1* clinical variants on mitochondria-lysosome membrane contacts sites’, published in BiO. Lara is a postdoc in the Neurogenetics and Molecular Medicine lab of Dr Francesc Palau and Dr Janet Hoenicka at Sant Joan de Déu Research Institute (IRSJD), Barcelona, Spain, investigating the pathophysiology of peripheral neuropathies and new therapeutic targets.


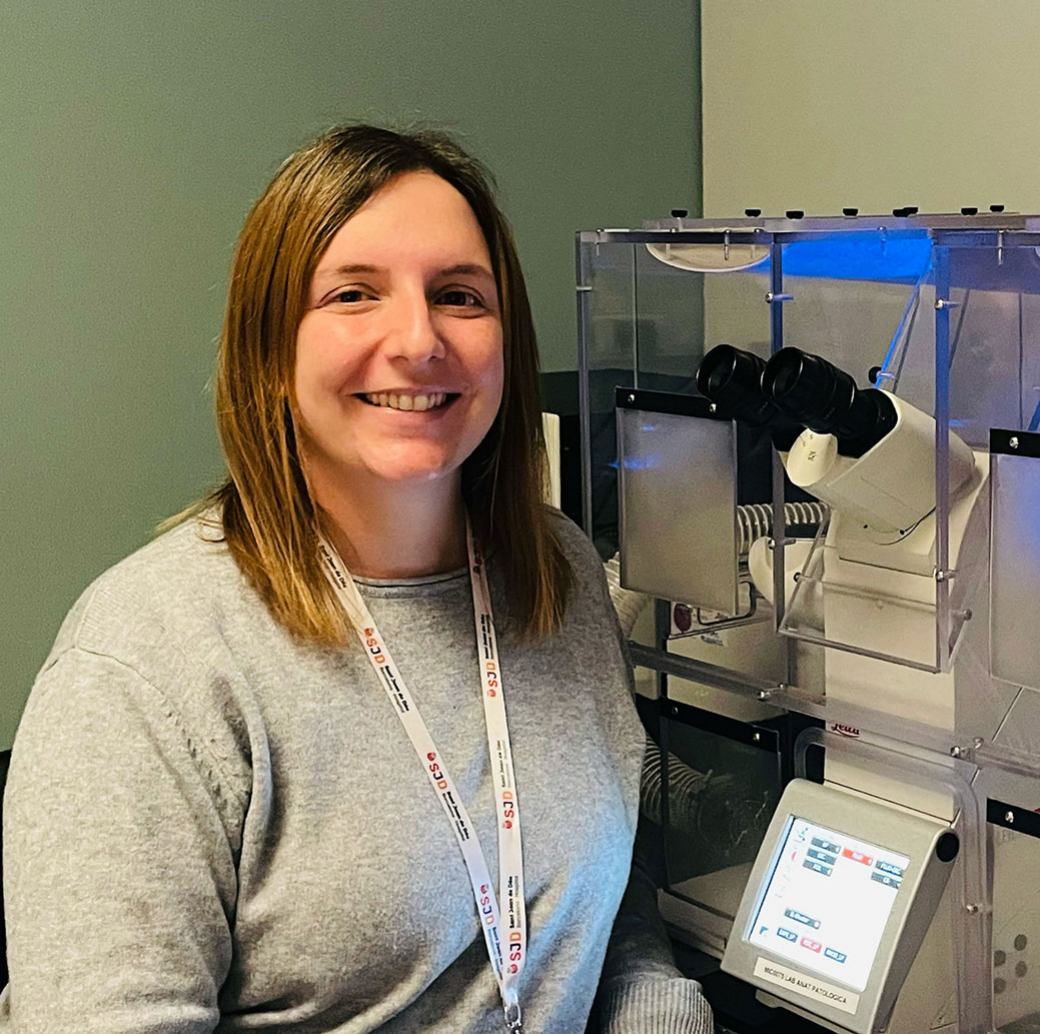


**Lara Cantarero**

**Describe your scientific journey and your current research focus**

I did my bachelor's degree in biology at the University of Barcelona. In 2010 I started my PhD at the Cancer Research Center of Salamanca (IBMCC-CIC), in Dr Lazo's group, where I characterized the molecular mechanism by which VRK1 kinase regulates the dynamics and stability of Cajal bodies. In 2016, I joined the Neurogenetics and Molecular Medicine group as a postdoctoral researcher, directed by Drs Palau and Hoenicka at the Sant Joan de Déu Research Institute (IRSJD). My research has been focused on the pathophysiology of Charcot-Marie-Tooth neuropathy, in cellular and murine models, patient fibroblasts, and induced neurons. In recent months, I have joined the Translational Diagnostic and Therapeutic Program (TDTP) of our laboratory for translational research in patient samples and the search for therapeutic targets. The TDTP objective is to use molecular and cellular biology to explore new treatments and clinical trials, through molecular characterization of patients, identification of cell phenotypes and biomarkers, drug screening for repositioning and personalized molecular therapies.

**Who or what inspired you to become a scientist?**

Cellular biology has fascinated me since childhood. Science is a way of thinking, and I think you are truly privileged if you ask the right question so that the answer comes back meaningful. And then, if nature tells you the answer, it's something magical!

As the scientist Carl Sagan, whom I have always admired, wrote:

*Science is part and parcel humility. Scientists do not seek to impose their needs and wants on Nature, but instead humbly interrogate Nature and take seriously what they find. We are aware that revered scientists have been wrong. We understand human imperfection. We insist on independent and – to the extent possible – quantitative verification of proposed tenets of belief. We are constantly prodding, challenging, seeking contradictions or small, persistent residual errors, proposing alternative explanations, encouraging heresy. We give our highest rewards to those who convincingly disprove established beliefs.*

**How would you explain the main finding of your paper?**

Charcot-Marie-Tooth neuropathy is a rare disease, although it is the most common hereditary neuropathy, affecting 2.8 million people worldwide. Currently, there is no effective treatment. It is for this reason that the study of the affected cellular processes is necessary to find out possible new therapeutic targets that help in the development of advanced therapies. In this work, we have delved into one of the cellular processes altered in Charcot-Marie-Tooth caused by mutations in the *GDAP1* gene: the mitochondria–lysosome membrane contact sites, a microenvironment where many cellular processes are altered in patients.

**What are the potential implications of this finding for your field of research?**

I hope that these results will bring more functional knowledge to the field of Charcot-Marie-Tooth research. Specifically, in *GDAP1*-related Charcot-Marie-Tooth, which can be very disabling. In addition, due to the very nature of GDAP1, an atypical GST-transferase, the results begin to shed light on the relevance of these proteins in the structure of the mitochondrial–lysosome contacts.

**Figure:**
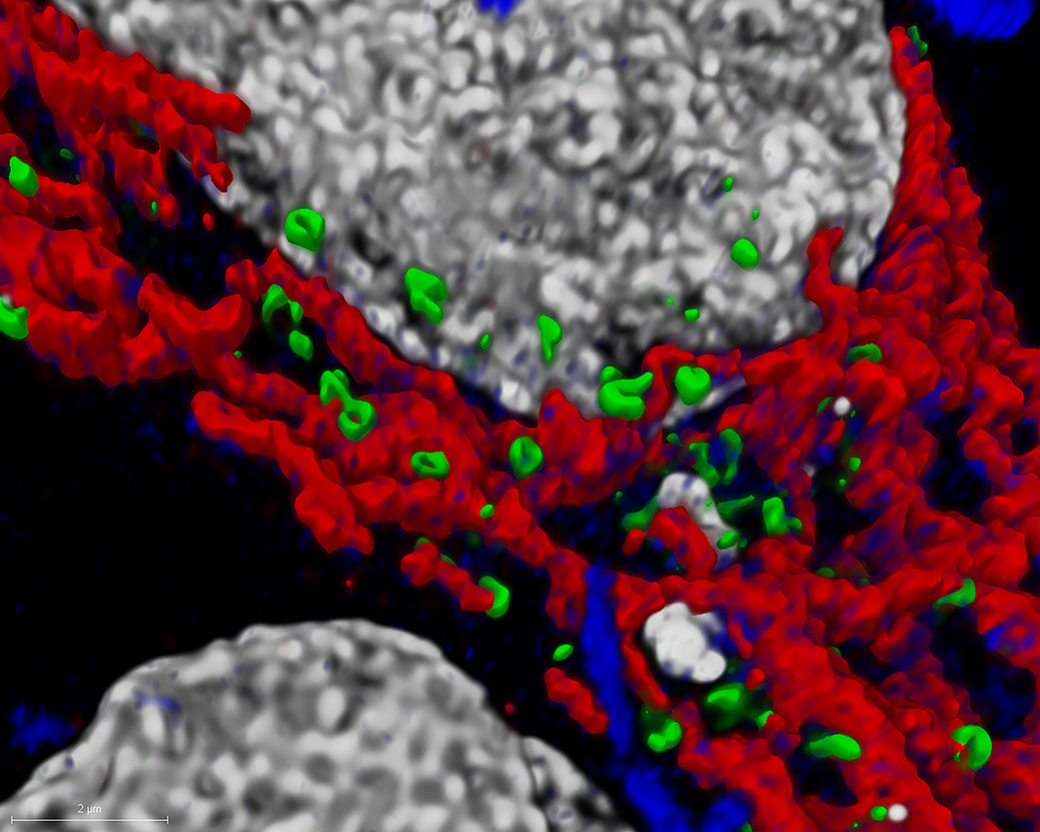
Three-dimensional confocal image projection of GDAP1 protein (red), mitochondria (blue) and lysosomes (green) in neuroblastoma SH-SY5Y cell line.

**Which part of this research project was the most rewarding?**

Charcot-Marie-Tooth neuropathy due to *GDAP1* mutations presents differences in clinical expression in relation to the Mendelian inheritance pattern: the recessive forms are very severe while the dominant forms are mild or moderate. The fact that we were able to show a correlation between the Mendelian pattern of inheritance and the different consequences on mitochondrial network morphology and mitochondria-lysosome contacts was remarkable. These results also suggest that, even for the same gene, the design of personalized therapies can vary to rescue the underlying defect at its source.

**What do you enjoy most about being an early-career researcher?**

Personally, I think it is a wonderful stage in my scientific career. After more than 10 years of dedicating myself to science, I have been learning a lot of techniques, acquiring great autonomy and, above all, starting to enjoy every question and answer achieved. You no longer have the rush and the time pressure of the doctoral thesis, but you can go driving a project, thinking about future experiments, and achieving major milestones. In addition, I love to be able to teach the younger researchers and students who come to the lab, to see their faces when they get their first western blot and to be able to transmit and spread them with a passion for science.

**What piece of advice would you give to the next generation of researchers?**

Starting a scientific career can be difficult at first, you will spend many hours in the lab. That's why I think it's very important for young scientists to be as passionate and inspired as possible. The life of a scientist is full of sacrifices, obstacles, experiments that do not work out and frustrations, however, all these difficulties are compensated by a fascinating side, when you confirm your hypothesis, when you get that experiment you have been struggling with for months, or when you identify the key cellular process that will lead to future therapies. I can only say that science gives you much more than it takes away. It is also crucial to have a great mentor, someone who orientates you, gives advice and helps you to grow.

**What's next for you?**

I have enjoyed doing science for more than 10 years. I still enjoy working at my bench, doing experiments, and writing papers and research projects. I hope I can continue to do this for a very long time. In the coming years, I will focus on the study and design of new personalized therapies in pediatric rare diseases, a research area where cell biology and clinical biology meet, in a round-trip path from the laboratory and the cell to the patient.
